# Regulating Factors of PrP^res^ Glycosylation in Creutzfeldt-Jakob Disease - Implications for the Dissemination and the Diagnosis of Human Prion Strains

**DOI:** 10.1371/journal.pone.0002786

**Published:** 2008-07-30

**Authors:** Etienne Levavasseur, Isabelle Laffont-Proust, Émilie Morain, Baptiste A. Faucheux, Nicolas Privat, Katell Peoc'h, Véronique Sazdovitch, Jean-Philippe Brandel, Jean-Jacques Hauw, Stéphane Haïk

**Affiliations:** 1 INSERM, Avenir Team - Human Prion Diseases, Paris, France; 2 APHP, R. Escourolle Neuropathology Laboratory, Paris, France; 3 InVS, French National Center of Reference for Unconventional Transmissible Agents, Paris, France; 4 Biochemistry and Molecular Biology Department, Lariboisière Hospital, Paris, France; Institute of Neuropathology, Switzerland

## Abstract

**Objective:**

The glycoprofile of pathological prion protein (PrP^res^) is widely used as a diagnosis marker in Creutzfeldt-Jakob disease (CJD) and is thought to vary in a strain-specific manner. However, that the same glycoprofile of PrP^res^ always accumulates in the whole brain of one individual has been questioned. We aimed to determine whether and how PrP^res^ glycosylation is regulated in the brain of patients with sporadic and variant Creutzfeldt-Jakob disease.

**Methods:**

PrP^res^ glycoprofiles in four brain regions from 134 patients with sporadic or variant CJD were analyzed as a function of the genotype at codon 129 of *PRNP* and the Western blot type of PrP^res^.

**Results:**

The regional distribution of PrP^res^ glycoforms within one individual was heterogeneous in sporadic but not in variant CJD. PrP^res^ glycoforms ratio significantly correlated with the genotype at codon 129 of the prion protein gene and the Western blot type of PrP^res^ in a region-specific manner. In some cases of sCJD, the glycoprofile of thalamic PrP^res^ was undistinguishable from that observed in variant CJD.

**Interpretation:**

Regulations leading to variations of PrP^res^ pattern between brain regions in sCJD patients, involving host genotype and Western blot type of PrP^res^ may contribute to the specific brain targeting of prion strains and have direct implications for the diagnosis of the different forms of CJD.

## Introduction

Human prion diseases are fatal neurodegenerative diseases. They are clinically characterized by progressive dementia associated with various neurological symptoms and neuropathologically by spongiosis, gliosis and neuronal loss in the brain. An almost constant biochemical hallmark is the accumulation of an abnormal partially protease-resistant isoform (PrP^res^) of the host-encoded cellular prion protein (PrP^c^). According to the protein only hypothesis, the mechanism for prion propagation is thought to involve the conversion of PrP^c^ into PrP^res^ [1], [2]. In humans, prion disorders occur in acquired, inherited and sporadic forms. Sporadic Creutzfeldt-Jakob disease (sCJD) accounts for more than 80 percent of the cases [3]. Among sCJD patients, a wide range of clinical and neuropathological phenotypes is observed. Recently, efforts have focused on the molecular basis of such phenotypical diversity considering the biochemical properties of PrP^res^ and the presence of polymorphisms within the gene encoding PrP (*PRNP*). Indeed, a classification of PrP^res^ Western blot patterns, obtained after proteinase K (PK) digestion which leads to PK-resistant core fragments of either 21 kDa (type 1) or 19 kDa (type 2), has been proposed [4]. This difference results from distinct PK cleavage sites of PrP^res^ [5]. Another contribution to the sCJD heterogeneity is the methionine/ valine polymorphism at the codon 129 of *PRNP* [6]. By correlating codon 129 genotypes and PrP^res^ types to clinical and pathological features in a series of 300 sporadic CJD, six molecular combinations corresponding to some phenotypic variants were identified [7]. However, these observations are based on the postulate that the same PrP conformer accumulates in the whole brain [8]. This has been recently questioned since more than one PrP^res^ type in the brain from sCJD patients has been observed [9]. Human prion protein has two sites of N-glycosylation at residues 181 and 197 [10]. Depending on the occupation of these sites, different glycoforms are identified on Western blot, the proportion of which may vary between prion strains. For example, a higher amount of diglycosylated PrP^res^ (type 2B) distinguishes vCJD from sCJD patients [8]. This particular pattern is retained when the type 2B prion strain is transmitted from one species to another. This contributed to the identification of the BSE as the origin of vCJD epidemics in humans, suggesting that protein glycosylation may be part of specific strain properties [11]. Glycosylation seems also to be involved in the targeting of brain regions by prions leading to strain-specific lesion profiles as previously suggested in a study using transgenic mice overexpressing hamster PrP mutated at the 2^nd^ glycosylation site [12]. Other results recently obtained using gene-targeted mice showed also that N-linked glycosylation of the prion protein influences lesion profile and plaque occurrence induced by some experimental strains of scrapie [13]. While PrP^res^ glycosylation is a key factor in strain diagnosis and probably brain targeting in human diseases, whether it is regulated between brain regions, and how, remains largely unknown.

Hence, we analyzed the pattern of PrP^res^ glycosylation from four different brain regions in a large series of 134 patients with CJD and investigated how brain region, *PRNP* genotype at codon 129 and PrP^res^ type may affect the accumulation of PrP^res^ glycotypes.

## Results

### Classification of CJD cases

**Figure 1:**
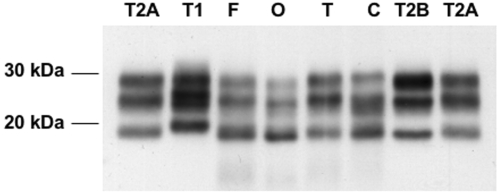
Western blot of PrP^res^ in different brain regions of a patient with sporadic Creutzfeldt-Jakob disease using the 3F4 reference antibody. Type of PrP^res^ in frontal cortex (F), occipital cortex (O), thalamus (T) and cerebellum (C) was determined using type 1, type 2A and type 2B standards after PK digestion.

Using results from Western blot typing of PrP^res^ and *PRNP* genotyping, we obtained a distribution of the different molecular subtypes of sCJD cases with a predominance of MM1 and VV2 patients (Fig 1 and Table 1), consistent with the distribution reported by Head et al. in a UK population [8]. All the patients with vCJD were methionine homozygotes and had type 2B PrP^res^.

**Table 1:** Description of sporadic and variant Creutzfeldt-Jakob disease populations.

Type	MM	MV	VV	Total
sCJD				
Type 1	70 (56,9)	8 (6,5)	1 (0,8)	79 (64,2)
Type 2A	3 (2,4)	18 (14,6)	23 (18,7)	44 (35,8)
Total	73 (59,4)	26 (21,8)	24 (19,5)	123 (100)
vCJD				
Type 2B	11 (100)	0	0	11 (100)

Number of sporadic CJD (sCJD) cases and variant CJD (vCJD) cases are distributed according to the genotype at codon 129 of *PRNP* (MM: methionine homozygote, VV: valine homozygote, MV: methionine-valine heterozygote) and PrP^res^ type (type 1, type 2A or type 2B). Absolute numbers of cases are indicated and the percentage of total is shown in round brackets.

### Regional variations of PrP^res^ glycoform ratio in sCJD patients

#### Frontal cortex versus occipital cortex

**Figure 2:**
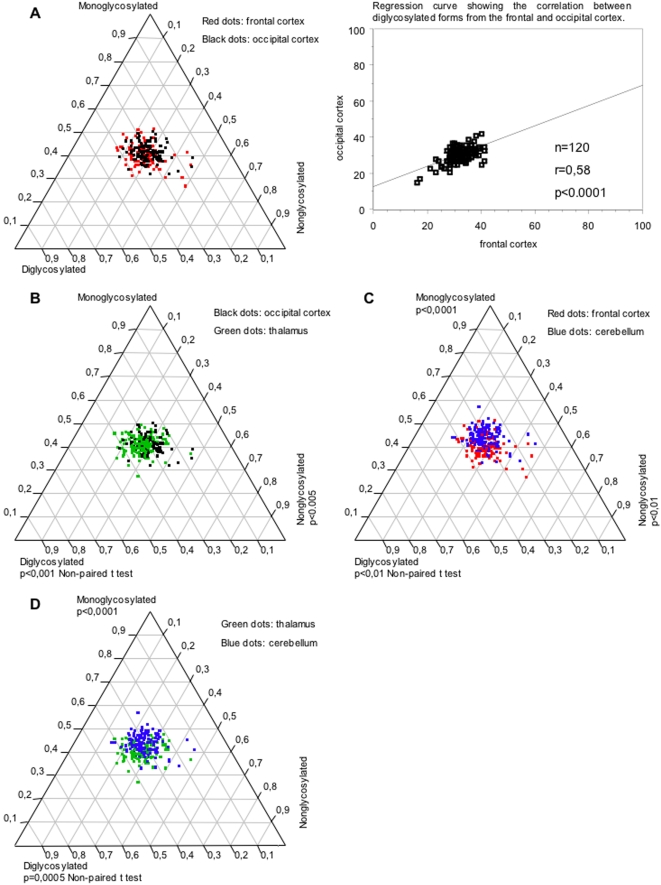
Regional variations of PrP^res^ glycoforms in brains from sCJD patients. PrP^res^ glycoform ratios from the frontal and occipital cortex were not statistically different (A, left panel). Each dot represents one brain area from a patient and is defined on the ternary plot by three coordinates corresponding to the relative intensity of di-, mono- and nonglycosylated bands. The correlation between diglycosylated forms from these two regions is shown on the regression curve (A, right panel). The thalamus exhibited more diglycosylated forms and less nonglycosylated forms than the occipital cortex (B). The cerebellum exhibited more monoglycosylated forms than the cortical regions (C). More diglycosylated forms were also detected in the thalamus when compared with the cerebellum, which showed more monoglycosylated forms (D).

No difference was observed between these two regions as illustrated on Fig 2A, left panel. The three glycoforms were evenly distributed in both regions. The proportion of di- and nonglycosylated forms in the frontal cortex correlated well with those observed in the occipital cortex (regression analysis: r = 0,58) (Fig 2A, right panel).

#### Frontal or occipital cortex versus thalamus

PrP^res^ glycoform ratios from the frontal cortex and the thalamus were not statistically different. However, they differed significantly between the occipital cortex and the thalamus, which showed more diglycosylated forms than the occipital cortex (Fig 2B).

#### Frontal or occipital cortex versus cerebellum

Compared to the frontal and the occipital isocortices, a higher proportion of monoglycosylated forms was found in the cerebellum (Fig 2C). Frontal cortex exhibited more diglycosylated and nonglycosylated forms than the cerebellum.

#### Cerebellum versus thalamus

Higher amounts of diglycosylated forms were detected in the thalamus while an increased accumulation of monoglycosylated forms occurred in the cerebellum (Fig 2D).

### Regional variations of PrP^res^ glycoform ratio in vCJD patients

In the 11 French vCJD patients that we studied, we consistently observed the typical type 2B PrP^res^ profile whithout any significant difference between brain regions.

### Influence of the PrP genotype at codon 129 on PrP^res^ glycoform ratio

#### MM genotype

**Figure 3:**
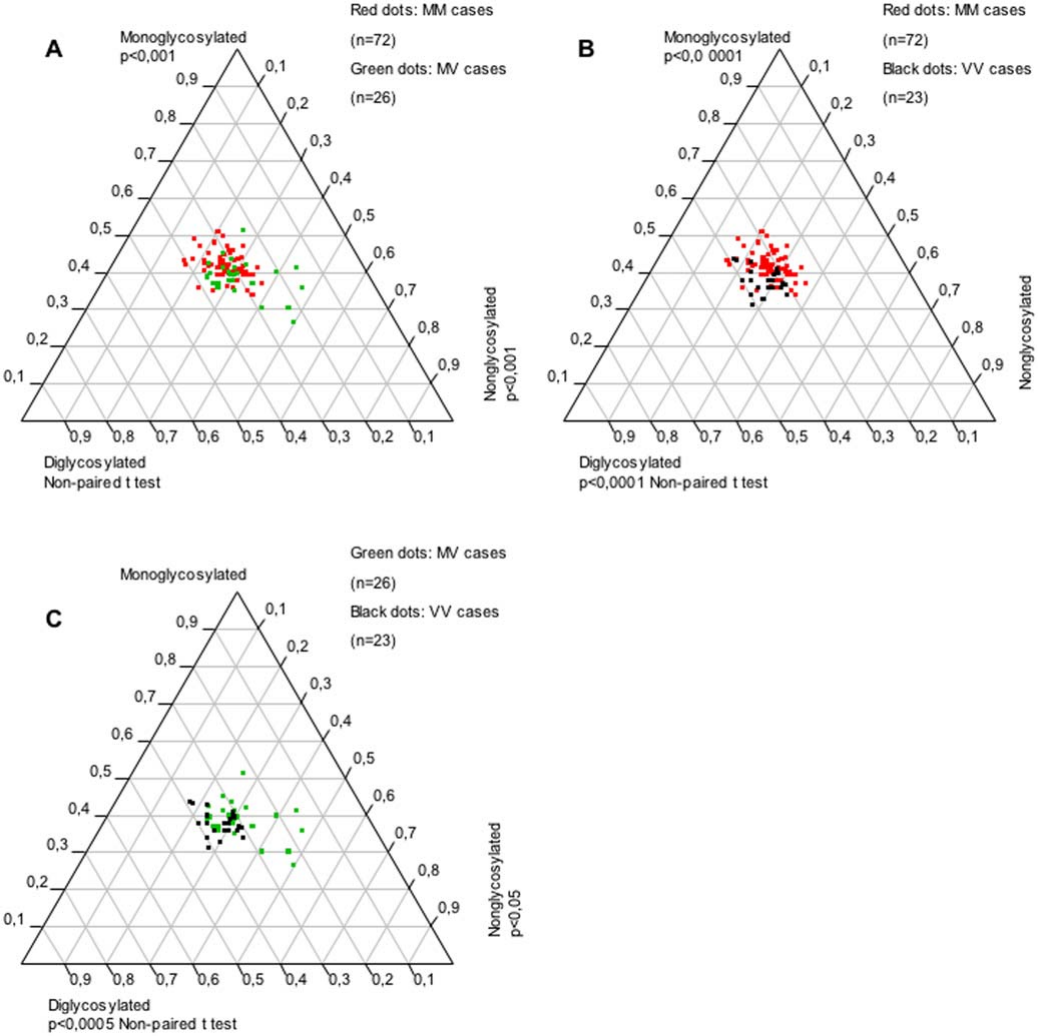
Influence of *PRNP* codon 129 genotype on PrP^res^ glycoform ratios in the frontal cortex from sCJD patients. Each dot represents one brain area from a patient and is defined on the ternary plot by three coordinates corresponding to the relative intensity of di-, mono- and nonglycosylated bands. More monoglycosylated forms were detected in methionine homozygote patients compared to other genotypes (A, B). Methionine/ valine heterozygote patients showed more nonglycosylated forms (A, C). Valine homozygote patients had more diglycosylated forms (B, C).

A significant increase of monoglycosylated forms was observed in the frontal cortex (Fig 3A, 3B), occipital cortex (Fig S1A, S1B), thalamus (Fig S2A, S2B) and cerebellum (Fig S3A, S3B), compared to MV and VV genotypes.

#### MV genotype

Frontal cortex (Fig 3A, 3C), occipital cortex (Fig S1A, S1C), thalamus (Fig S2A, S2C) and cerebellum (Fig S3A, S3C) from MV cases accumulated significantly more nonglycosylated forms than MM and VV cases.

#### VV genotype

In the frontal cortex (Fig 3B, 3C), thalamus (Fig S2B, S2C) and cerebellum (Fig S3C) from VV patients, diglycosylated forms were significantly more abundant compared to MV patients and MM patients.

Therefore, a tendency was observed for each genotype to favour the accumulation of one glycoform in the frontal cortex, the occipital cortex, the thalamus and, to a lesser extent, in the cerebellum. Valine homozygosity favoured the diglycosylated form, methionine homozygosity favoured the monoglycosylated form, and heterozygosity favoured the nonglycosylated form.

### Influence of the PrP^res^ type on glycoform diversity

**Figure 4:**
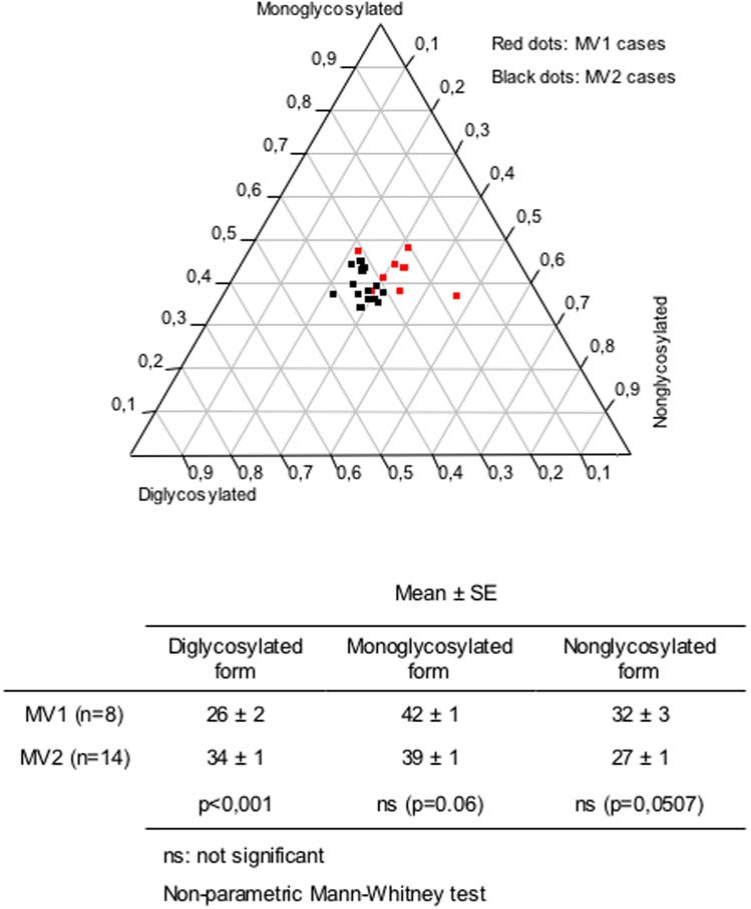
Influence of the PrP^res^ type on PrP^res^ glycoforms in the thalamus. Each dot represents one brain area from a patient and is defined on the ternary plot by three coordinates corresponding to the relative intensity of di-, mono- and nonglycosylated bands. To rule out any codon effect, the influence of PrP^res^ type was assessed in MV1 and MV2 patients. These two populations are almost separated on the ternary plot. A higher content of diglycosylated forms is observed in the heterozygote MV2 subgroup compared to the MV1 subgroup.

We found that the type of PrP^res^ affected the pattern of PrP^res^ glycosylation in the thalamus, while little or no effect was detected in the other studied brain regions. Two distinct populations corresponding to MV1 and MV2 subgroups were evidenced. Subgroup MV2 had more diglycosylated forms and subgroup MV1 presented more nonglycosylated forms (Fig 4).

**Figure 5:**
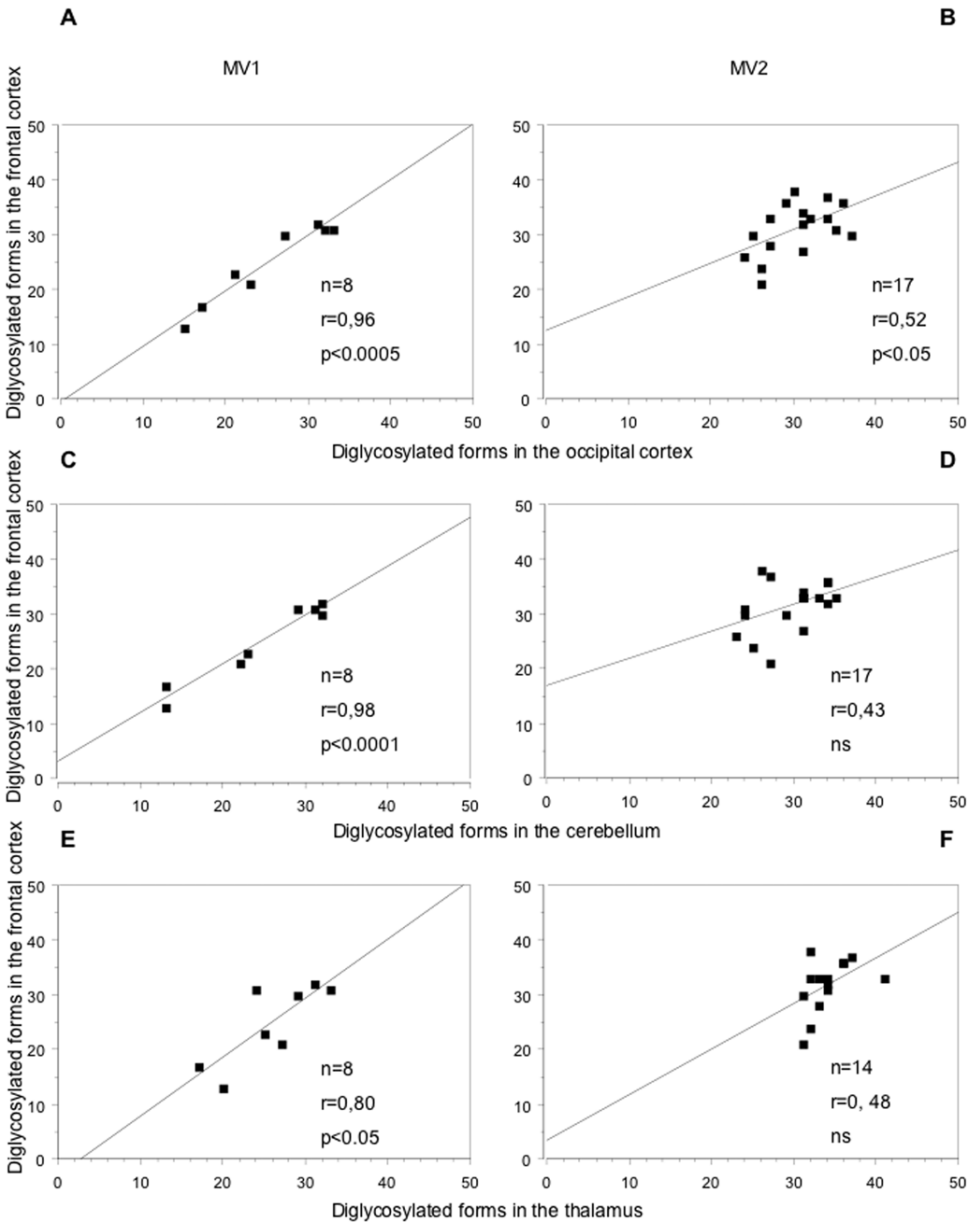
Influence of the PrP^res^ type on PrP^res^ glycoforms. A strong correlation is observed between diglycoforms of type 1 PrP^res^ from different brain regions (r values equal to or above 0.80) (A, C, E). On the contrary, the glycoform ratio varies to a larger extent when patient had type 2 PrP^res^ (r values equal to or below 0.52) (B, D, F).

In addition, the homogeneity of PrP^res^ glycoform pattern between regions dramatically varies depending on PrP^res^ types. Indeed, regression analysis demonstrated r values equal to or above 0,80 in MV1 group (Fig 5A, 5C, 5E) when correlating diglycosylated form ratio between regions, while r values from MV2 group ranged from 0,43 to 0,52 (Fig 5B, 5D, 5F). These lower correlations indicate a greater heterogeneity between glycoform ratio from different brain regions.

## Discussion

This study provides new insight into the analysis of PrP^res^ heterogeneity within sCJD cases, based upon the biochemical analysis of at least 3 different brain regions from 123 sCJD cases. This large series of sCJD tissues allowed us to identify the diversity of PrP^res^ glycoforms within sporadic CJD forms and to establish a correlation with *PRNP* genotype, brain region and Western blot type of PrP^res^. Our results demonstrate that the regional distribution of PrP^res^ glycoforms within one individual is heterogeneous in the sporadic form of CJD and suggest the involvement of precise regulating mechanisms.

Different factors may contribute to this regulation. In the normal brain of human, hamster and mouse, PrP^c^ glycosylation pattern is submitted to a regional diversity [14], [15], [16]. In infected human brains, the diversity of PrP^res^ glycoprofiles may result from such a regional variation of the substrate for prion replication. According to the prion hypothesis, this mechanism suggests that the glycoprofile of PrP^c^ influences the glycoform ratio of PrP^res^ in the conversion process. In acellular models of PrP conversion indeed, the use of PrP^c^ with modified glycoforms ratio partially modifies the glycosylation pattern of newly formed PrP^res^ [17]. In the present study, we observed that the genotype at codon 129 of *PRNP* influenced the glycoform ratio of PrP^res^ in a region dependent manner. This raises the possibility that the regional glycosylation state of PrP^c^ may be affected by *PRNP* polymorphisms. It may be of interest to document such a regulation in the normal human brain.

Several studies have pointed out a relation between PrP amino acid sequence (i.e. *PRNP* genotype) and PrP^res^ glycosylation in inherited diseases associated to mutation located close to (D178N, V180I, T183A, E200K) or remote (P102L) from the N-glycosylation sites [18], [19]. Whether the presence of a mutation may modify the glycoform ratio of PrP^c^ in the human brain is poorly documented. However we previously showed that in patients with the V180I mutation, no diglycosylated PrP^res^ is produced, while PrP^c^ glycosylation is normally processed [20]. This clearly indicates that additional factors, different from the glycosylation state of PrP^c^, may influence the glycoform ratio of the accumulated PrP^res^ in the pathological condition.

Type 1 or type 2 PrP^res^ are thought to correspond to distinct accessibilities of proteinase K to cleavage sites resulting from different conformations of PrP^res^. A diversity of glycotypes has been described for type 2 PrP^res^, linked to the various forms of the diseases (sporadic, variant CJD, familial fatal insomnia). In sporadic CJD patients with the same genotype at codon 129, we found that PrP^res^ type markedly influences the glycoform ratio of the accumulated PrP^res^ in definite brain regions. This suggests that some conformers are more prone to convert distinct glycoforms of PrP^c^ in sporadic diseases and that this phenomenon is regionally regulated. In addition, to explain the strain mutation phenomenon, it has been recently proposed that a single strain may consist of an ensemble of molecular species containing a dominant PrP^res^ type [21]. Such conformers may differ by the glycoform species they favor during replication. It can be speculated that regional factors influence the selection of PrP^res^ subpopulations leading to the variation of the glycoform ratio that we finally detected.

The regional variation of PrP^res^ glycoform ratio demonstrated in this study may be of interest in the understanding of “lesion profile” and brain targeting by different prion strains. The mechanisms that favor the accumulation of a given strain in a given brain region are not known, but the regulation of PrPc/ PrP^res^ glycosylation is likely involved in this process [12], [13], [22]. As a matter of fact, we found in sCJD cases that the thalamus favored the production of type 2 PrP^res^ presenting a high content in diglycosylated forms. It is noteworthy that besides sCJD, vCJD and FFI are associated with a high content of diglycosylated forms and are characterized by a “targeting” of the thalamus. In all studied brain areas from French vCJD patients, as already reported in the U.K. cases, we observed that the glycoform ratio was remarkably constant. In this infectious form of prion disorders, the pathogenic events are initiated by an exogenous PrP^res^ from contaminated bovine tissues with its own defined characteristics, such as a high glysosylated content that is maintained through interspecies transmission. By contrast, the causative event in sCJD is thought to be a rare, spontaneous and endogenous conversion of the host-encoded PrP^c^ into PrP^res^. The diversity of glycoform ratios that we observed in sCJD may reflect the regulation of an endogenous phenomenon by various host factors (genotype, brain region) that may be overwhelmed in the case of an infection by a virulent prion strain such as the bovine agent in vCJD patients.

**Figure 6:**
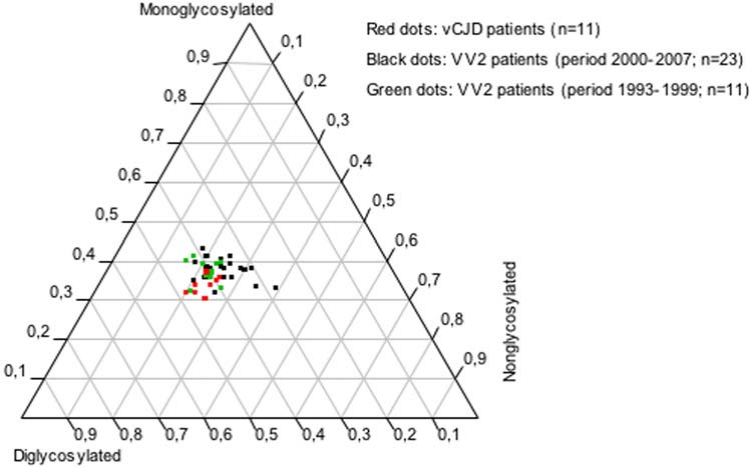
Similar PrP^res^ glycoform ratios with high glycosylation site occupancy in thalamus from VV2 sCJD patients and vCJD patients. Each dot represents one brain area from a patient and is defined on the ternary plot by three coordinates corresponding to the relative intensity of di-, mono- and nonglycosylated bands. We compared the VV2 patients from our sCJD series and vCJD patients with VV2 sCJD patients who died before or just after the first variant breakout in 1996. No difference was observed between VV2 patients from the 2000–2007 series and VV2 patients from the period 1993–1999.

Our observation of PrP^res^ glycoprofile diversity in sCJD raises the question of the strain diagnosis based on biochemical PrP^res^ analysis. The type 2B PrP^res^ profile is widely used as a diagnosis marker of definite vCJD that until now has been observed in MM patients only. However, in some VV2 CJD patients, the high proportion of diglycosylated PrP^res^ we observed in the thalamus was undistinguishable from the PrP^res^ profile detected in vCJD cases. Studies using transgenic mice expressing human PrP suggest that clinicopathological and biochemical presentations of BSE infection may vary in humans with the genotype at codon 129 [23]. One possible explanation of our findings in these particular VV2 cases could be BSE infection (i.e. vCJD) in valine homozygote patients. However, the mean age at death (>65 years, except one case who was 27) and the neuropathological pattern (absence of florid plaques) do not support such hypothesis. While the first MM vCJD patients occurred simultaneously in UK and France, the peak of French vCJD epidemics occurred in 2002 more than 5 years later than in the UK. Comparison of the 1980–1995 pattern of BSE exposition in the UK and France indicated that it peaked also later in France [24]. This probably explains the different temporal pattern of vCJD incidence. We analyzed retrospectively whether some French VV2 CJD patients who died between 1993 and 1999 exhibited also a thalamic PrP^res^ profile with high glycosylation site occupancy similar to what is observed in vCJD. The same profile of PrP^res^ ressembling type 2B could also be detected in patients who died during the 1993–1999 period (Fig 6). This suggests that these VV patients had indeed a genuine sporadic disease rather than an atypical vCJD, which is very unlikely to have occurred in an unrelated genotype and before 1996 in France.

**Figure 7:**
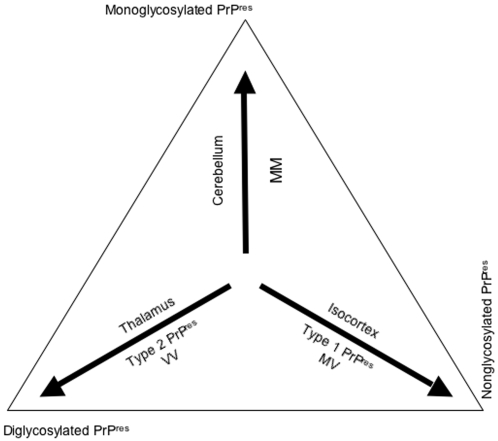
Schematic representation of regulation of PrP^res^ glycoforms by regional and molecular factors in sporadic Creutzfeldt-Jakob disease. Arrows indicate which glycoform of PrP^res^ is favored by individual factors. As an example, the accumulation of diglycosylated PrP^res^ is favored in the thalamus, in VV patients and in case of type 2 PrP^res^.

Finally, the present work evidences regulations that lead to highly significant variations of PrP^res^ pattern between brain regions in sCJD patients, involving host genotype and Western blot type of PrP^res^ as recapitulated in figure 7. These may contribute to the specific brain targeting of prion strains and have some diagnosis implications.

## Methods

### Case and tissue selection

This study was performed on patients referred to the French National Surveillance Network for CJD during the period january 2000 to march 2007 and for which clinical, genetic and neuropathological data were available to support a diagnosis of definite sCJD [25]. Only patients with at least three available regions out of the four studied (frontal cortex, occipital cortex, thalamus and cerebellar vermis) were considered. Patients with familial and iatrogenic CJD were excluded from the study. We included 123 sCJD cases (103 patients had 4 available regions), 55 male and 68 female subjects, and the mean age at death was 68 (range, 24–90 years). In addition, a group of 11 french vCJD cases was also studied, comprising of 4 male and 7 female subjects. The mean age at death was 36 (range, 20–58 years). The patients' relatives gave written informed consent for autopsy, and the study was designed in accordance with the relevant French legislation.

### PRNP analysis

DNA was extracted from buffy coat fraction of peripheral blood after obtaining written informed consent from patients. Codon 129 polymorphism of the *PRNP* gene and the absence of mutation or insertion was determined as described elsewhere [26].

### Biochemical analysis of PrP^res^

Frozen tissue was stored at −80°C. Brains were homogenized at 20% (w/v) using a Ribolyser (Bio-Rad) in 5% sterile glucose. One volume of a 20% NaCl solution and one volume of a detergent solution (20% sarkosyl, 2% SB3-14 and Tris 20 mM pH 7.4) were added to one volume of 20% brain homogenate. This mixture had a final pH of 7.6 and was digested with proteinase K at 10 µg/ml (diluted in Tris-HCl 50 mM pH 8, CaCl2 1 mM) for 1 h at 37°C. PrP^res^ was then purified by centrifugation at 27500 *g* for 2 h at room temperature on 20% sucrose cushion, according to the scrapie-associated fibril (SAF) protocol reported previously with slight modifications [27]. Samples were subjected to a 12% Tris-glycine sodium dodecyl sulfate-polyacrylamide gel electrophoresis (SDS-PAGE). Proteins were electroblotted onto nitrocellulose membrane. PrP staining was performed using anti-PrP monoclonal antibody 3F4, epitope 109–112 of human PrP sequence (Signet, MA, USA) at a dilution of 1 to 50000, and chemiluminescence (ECL; RPN-2209; Amersham Biosciences, Freiburg, Germany) for the detection of peroxidase activity [28]. Goat anti-mouse secondary antibody horseradish peroxidase conjugated used was from Pierce Biotechnology (31430, Rockford, IL) and used at a dilution of 1 to 5000. Quantitation of PrP^res^ glycoforms was performed using a GS-800 calibrated densitometer and dedicated Quantity One software (Bio-Rad Laboratories) as described [8]. Values for all PrP^res^ bands were measured on blots with a non-saturated signal and calculated as a percentage of the total PrP^res^. When necessary, additional blots were performed with serially diluted samples to obtain a signal in a linear range. Type 1 PrP^res^ and type 2 PrP^res^ were characterized by a nonglycosylated band migrating at 21 kDa and at 19 kDa, respectively, as described by Parchi et al. [6]. Typical standards of type 1, type 2A and type 2B PrP^res^ were systematically included to facilitate Western blot typing of PrP^res^. Molecular weights were determined using a protein standard (Novex Sharp Standard, Invitrogen). Cases of sCJD were classified according to Parchi et al. that identified six molecular subtypes on the basis of the *PRNP* codon 129 polymorphism and PrP^res^ type [7]. Cases of sCJD for which both types of PrP^res^ were detected using 3F4 antibody in a same brain region or in different regions were excluded from the study [8], [9], [29].

### Statistical analysis

Linear regression, correlation and non parametric tests were carried out using StatView v4.0 (Abacus Concepts, Berkeley, CA). A *p* value of less than 0.05 was considered to be statistically significant. Glycoform ratios were visualized on ternary plot as originally described by Glatzel et al. [30]. Automated graphical representations were generated using JMP software v6.0.0 (SAS).

## Supporting Information

Figure S1 Influence of *PRNP* codon 129 genotype on PrP^res^ glycoform ratios in the occipital cortex from sCJD patients. This brain region was less affected by genotype than the frontal cortex and the thalamus. However, methionine homozygote patients showed more monoglycosylated forms (A, B) and methionine/ valine heterozygote patients were associated with a predominance of nonglycosylated forms (A, C).
(3.75 MB TIF)

Figure S2 Influence of *PRNP* codon 129 genotype on PrP^res^ glycoform ratios in the thalamus from sCJD patients. Marked differences were observed in this brain region. More monoglycosylated forms were detected in methionine homozygote patients compared to other genotypes (A, B,). Methionine/ valine heterozygote patients presented more nonglycosylated forms (A, C). Valine homozygote patients had more diglycosylated forms (B, C).
(3.81 MB TIF)

Figure S3 Influence of *PRNP* codon 129 genotype on PrP^res^ glycoform ratios in the cerebellum from sCJD patients. Like the occipital cortex, this brain region was less affected by genotype than the frontal cortex and the thalamus. However, methionine homozygote patients remained associated with more monoglycosylated forms (A, B), methionine/ valine heterozygote patients were associated with a predominance of nonglycosylated forms (A, C), while valine homozygote patients clearly presented more diglycosylated forms (B, C).
(3.81 MB TIF)
