# Correction: Regulating Factors of PrP^res^ Glycosylation in Creutzfeldt-Jakob Disease - Implications for the Dissemination and the Diagnosis of Human Prion Strains

**DOI:** 10.1371/annotation/4d3f48de-5ff4-4442-a041-0e9c78c2049d

**Published:** 2008-09-16

**Authors:** Etienne Levavasseur, Isabelle Laffont-Proust, Émilie Morain, Baptiste A. Faucheux, Nicolas Privat, Katell Peoc'h, Véronique Sazdovitch, Jean-Philippe Brandel, Jean-Jacques Hauw, Stéphane Haïk

Etienne Levavasseur and Isabelle Laffont-Proust contributed equally to this work.

