# Supplementary figures and images for: Regulating Factors of PrPres Glycosylation in Creutzfeldt-Jakob Disease - Implications for the Dissemination and the Diagnosis of Human Prion Strains
Source: PLoS One. 2008 Jul 30;3(7):e2786. doi: 10.1371/journal.pone.0002786 (PMC2464735; doi:10.1371/journal.pone.0002786)

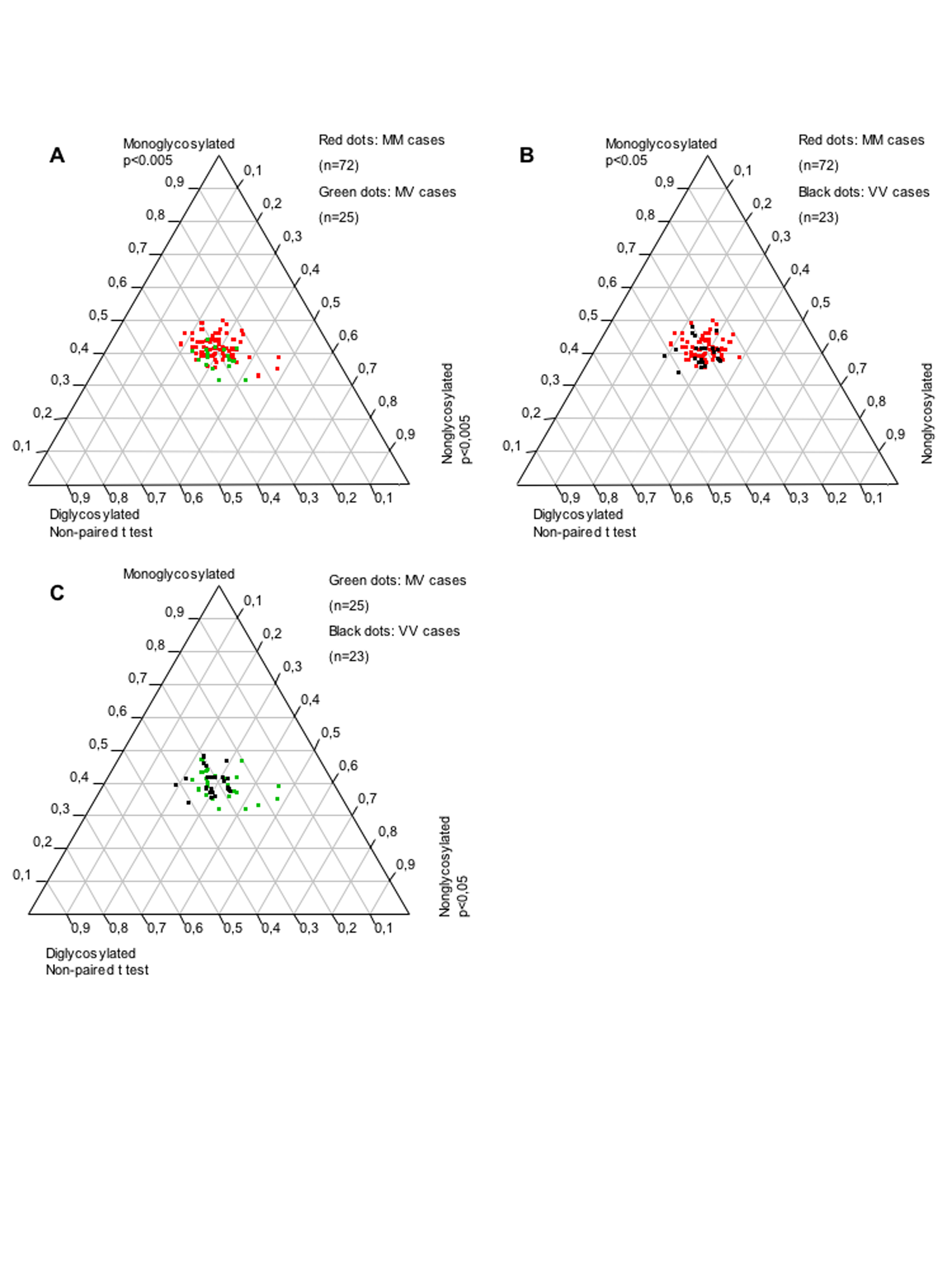

Supplement: Figure S1 — Influence of PRNP codon 129 genotype on PrPres glycoform ratios in the occipital cortex from sCJD patients. This brain region was less affected by genotype than the frontal cortex and the thalamus. However, methionine homozygote patients showed more monoglycosylated forms (A, B) and methionine/ valine heterozygote patients were associated with a predominance of nonglycosylated forms (A, C). (3.75 MB TIF) [file pone.0002786.s001.tif]

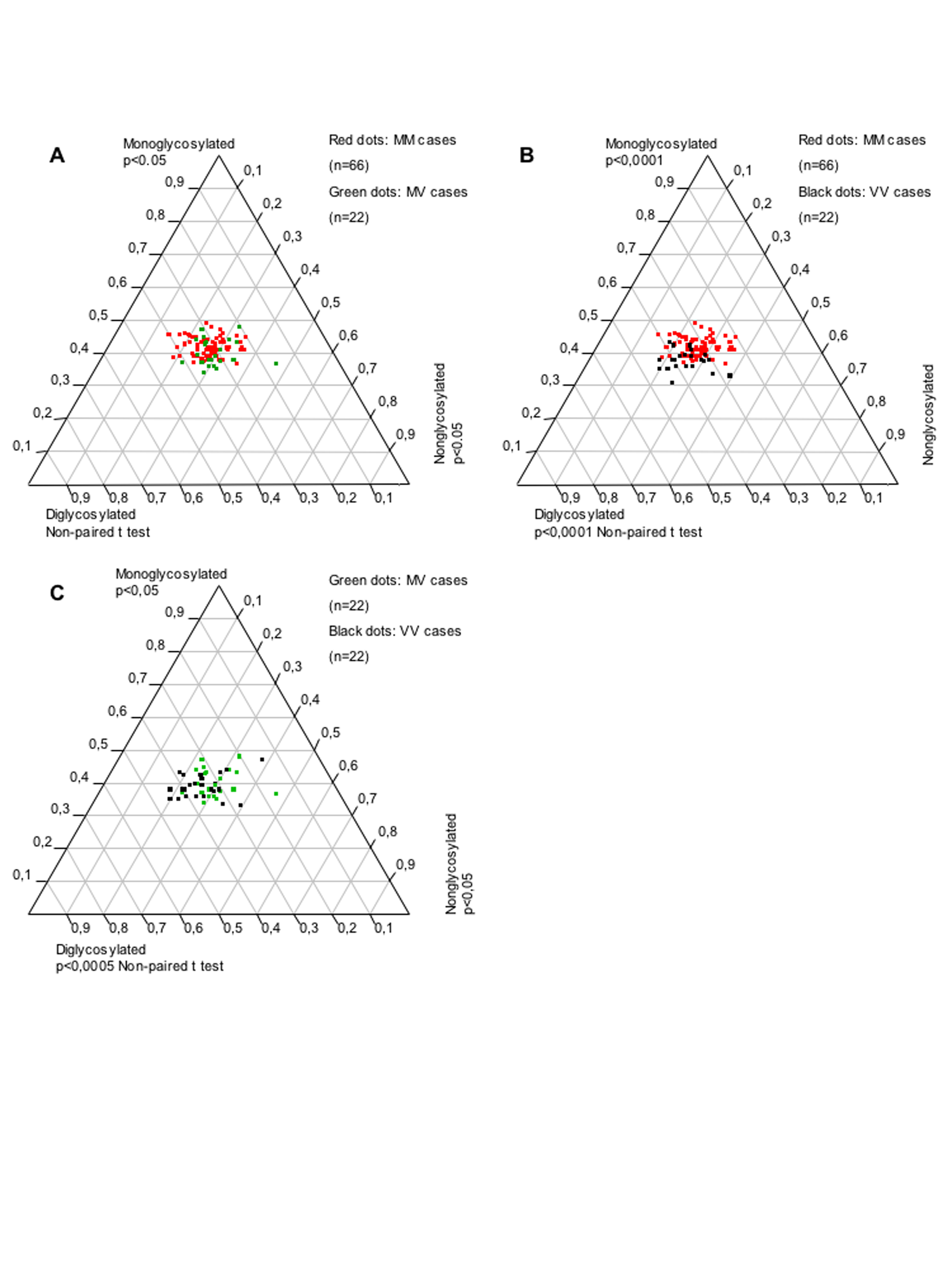

Supplement: Figure S2 — Influence of PRNP codon 129 genotype on PrPres glycoform ratios in the thalamus from sCJD patients. Marked differences were observed in this brain region. More monoglycosylated forms were detected in methionine homozygote patients compared to other genotypes (A, B,). Methionine/ valine heterozygote patients presented more nonglycosylated forms (A, C). Valine homozygote patients had more diglycosylated forms (B, C). (3.81 MB TIF) [file pone.0002786.s002.tif]

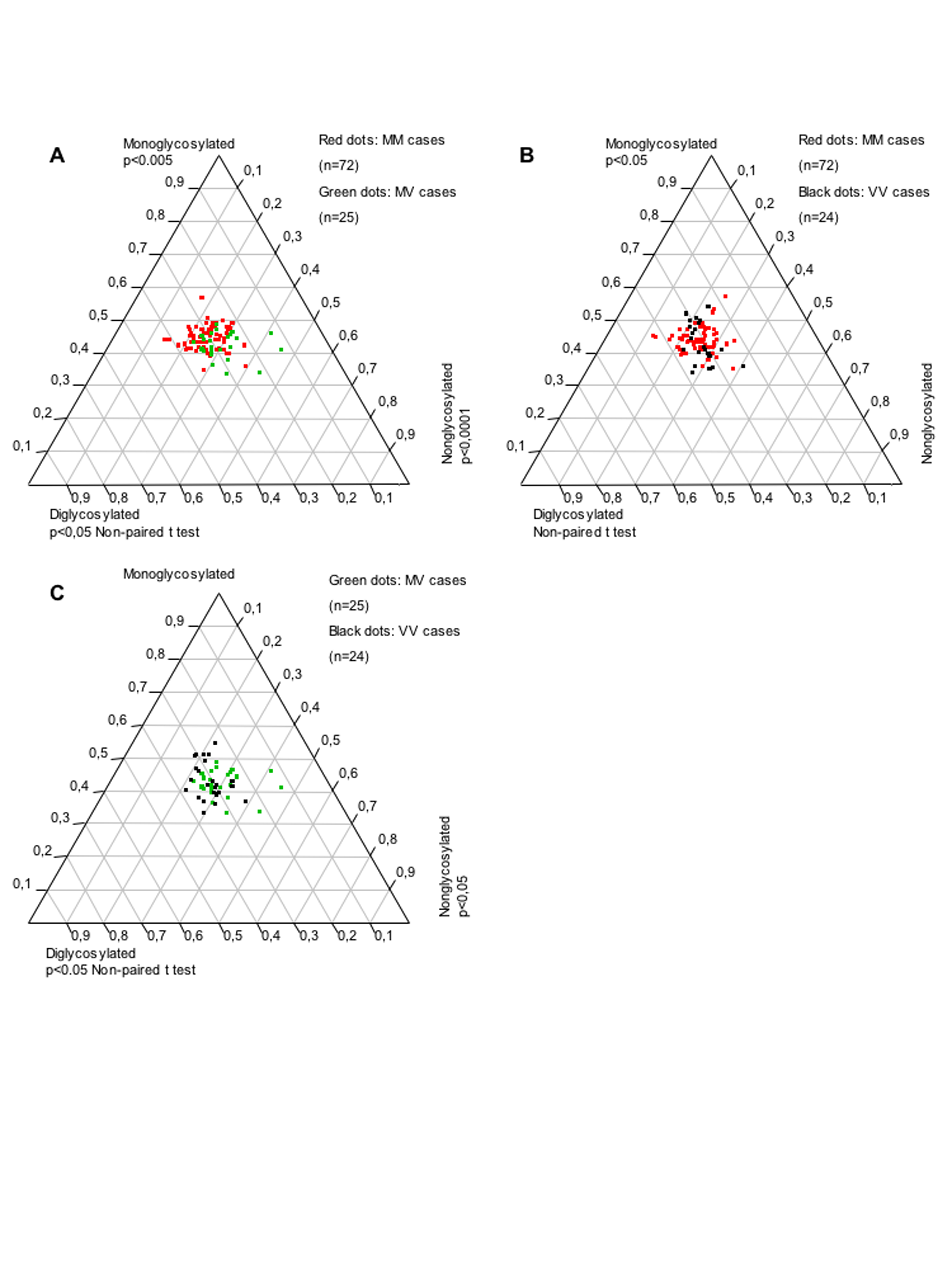

Supplement: Figure S3 — Influence of PRNP codon 129 genotype on PrPres glycoform ratios in the cerebellum from sCJD patients. Like the occipital cortex, this brain region was less affected by genotype than the frontal cortex and the thalamus. However, methionine homozygote patients remained associated with more monoglycosylated forms (A, B), methionine/ valine heterozygote patients were associated with a predominance of nonglycosylated forms (A, C), while valine homozygote patients clearly presented more diglycosylated forms (B, C). (3.81 MB TIF) [file pone.0002786.s003.tif]
